# Outcome Evaluation of a Short-Term Hospitalization and Community Support Program for People Who Abuse Ketamine

**DOI:** 10.3389/fpsyt.2018.00313

**Published:** 2018-07-17

**Authors:** Andrew M. H. Siu, Flora S. L. Ko, S. K. Mak

**Affiliations:** ^1^Department of Rehabilitation Sciences, Hong Kong Polytechnic University, Hong Kong, Hong Kong; ^2^North District Hospital, Hospital Authority of Hong Kong, Hong Kong, Hong Kong

**Keywords:** ketamine, drug misuse, outcome, evaluation, intervention, hospitalization, Chinese

## Abstract

Ketamine is a popular recreational drug among young people in Hong Kong. Long-term abuse of ketamine can lead to acute urological and medical issues, which often require immediate care at emergency rooms. Many patients require short-term hospitalization for medical management. This opens a brief time window, within which mental health professionals could engage young people who abuses ketamine in psychosocial, functional, and lifestyle interventions. The Crisis Accommodation Program (CAP) is a short-term hospitalization and community support program that addresses the health care needs of young people who abuse ketamine. During short-term hospitalization, the patient participates in a range of cognitive and psychosocial assessments, motivational interviewing, emotions management, and lifestyle re-design interventions. Upon discharge, social work professionals of non-government agencies continue to work with the patients on their action plans in the community. This evaluation study uses a quasi-experimental non-equivalent group design, in which the outcomes of the treatment group (*n* = 84) are compared with a comparison group (*n* = 34) who have a history of ketamine abuse but who have not joined the treatment program. The results confirm that the treatment group showed significant increases in motivation for treatment, reduction in drug use, improvement in cognitive screening tests, healthy lifestyle scores, and self-efficacy in avoidance of drugs over 13 weeks. When compared with the comparison group, the treatment group had significant decreases in anxiety and treatment needs and had moved from pre-contemplation to the contemplation or preparation stage. However, there were no significant changes in outcome measures covering lifestyle or self-efficacy in drug avoidance. Overall, the CAP is effective in reducing drug use, anxiety, and helping patients to move from pre-contemplation to the contemplation or preparation stage of change. The study results suggest that health care professionals can successfully engage young people who abuse ketamine to participate in a package of psychosocial interventions, motivational interviewing, and lifestyle re-design during their hospital stay for management of urological problems. The CAP also highlights the importance of collaboration between hospitals and community social services in the management of addiction.

## Introduction

Ketamine has long been a popular recreational drug used by young people in Hong Kong. The frequency of ketamine abuse has greatly increased from <1% in 1999 to becoming the most commonly abused drug in 2011 (1). Although the use of ketamine among young people has gradually decreased since 2011, there is a sizeable group of young people who have abused ketamine for many years. In 2015, ketamine remained the third most commonly abused drug, after heroin and methamphetamine (2).

The literature on the potential harm of long-term ketamine abuse is well documented. It has a negative impact on cognitive function including verbal memory, verbal learning, visual recognition difficulties, processing speed, and logical deduction (3). It is also linked to an increase in the incidence of mental illness such as depression and psychosis (4, 5). In addition, ketamine use is associated with risk-taking behavior like unsafe sex practices, participation in dangerous activities, and accidental death (6).

In recent years in Hong Kong, there has been a surge of young people visiting emergency rooms for a range of medical problems associated with ketamine use. These medical problems include acute urological problems such as small painful bladder, ureteric obstruction (7–9), papillary necrosis, and hepatic dysfunction (10), they also appear with symptoms of ketamine cystitis (like dysuria, urgency, chronic abdominal pain), and nasal problems (such as septal perforation) (11). Many urological symptoms could appear in several years after a period of intense Ketamine abuse (12, 13). Many patients need to stay in hospital for anything from one to a few weeks for treatment by a urologist or other medical specialist. The short stay in hospital for this group of young people opens a brief time window, within which mental health professionals can engage young people who abuse ketamine in psychosocial, functional, and lifestyle interventions. Upon discharge from the in-patient program, social work professionals can continue to support the patient and engage them in social and peer support programs in the community.

The North District Hospital of the Hospital Authority in Hong Kong designed a short-term hospitalization and support service called the CAP that addresses the health care needs of young people who abuse ketamine. The CAP has two key objectives: (1) To address the challenges of substance use and co-occurring medical, mental health, and lifestyle problems of the ketamine abusers, (2) To ensure the continuity of care for ketamine abusers by building a territory-wide collaborative model between hospitals and social service agencies in the community.

The collaborative service model of the CAP has five characteristics that aim to address the needs of “hidden” ketamine abusers in the community. First, the program uses a range of out-reach strategies to identify and recruit participants from the community. These strategies include social marketing through schools, referrals from social, and family services, and out-reach in the community. Some youth services also recruit participants by reaching them through the internet through data mining strategies. This last strategy addresses the phenomenon of “hidden youth” who may be disengaged from school and social life. Second, we conduct regular joint pre-admission screening and orientation programs for potential participants. Health care professionals from the hospital and social work professionals from the referring social service agency jointly interview and assess potential participants. The preliminary assessment includes medical problems linked to ketamine abuse, history of substance abuse, lifestyle, and daily functioning. We also brief the patient on the expectations of the program and invite them to join it. We find that this pre-admission screening and orientation helps the patient to develop appropriate expectations about the program and commitment to join it. Third, we designed a short-term (5-day) hospitalization program that addresses the key medical and psychosocial issues of ketamine abusers. The patient participates in the assessment on cognitive function, hand function, mental well-being, and lifestyle. Mental health professionals present the evaluation results to the patients, to try to raise their awareness of the health crisis they are facing. The occupational therapist provides a daily program that includes elements of brief motivational interviewing (14, 15), emotions management, and lifestyle re-design (16). Fourth, the hospitalization service has developed a network of social service partners, who ensure a smooth transition of treatment between hospital and social services in the community. We conduct joint case consultations, regular coordination meetings, and meetings with patients and family, so that we can monitor case progress as a team. Last, when the patient is ready for discharge, the team will work with the patient to set up action plans regarding their role and functional performance such as engagement in education, employment, household duties, or social activities. Overall, the program aims to engage the patient in addiction treatment during their hospital stay for medical management of urological and medical problems. Based on the “Stage of Change” model (17), we expect that a significant proportion of patients will move from “Pre-contemplation” to the “Contemplation” or “Preparation” stages of change in addiction during the short-term hospitalization program. Social service agencies continue to monitor and engage the patient in pursuing the action plan set up before discharge from hospital.

## Methods

The objective of this study was to analyze the outcomes of a short-term hospitalization and support program for people who abuse ketamine. A quasi-experimental, non-equivalent group design was used in the evaluation of change in those who participated in the program (treatment group), vs. those who had a history of ketamine abuse (comparison group) but did not join any drug treatment program during the period of study.

### Participants

Primary care, social, and outreach social work services helped to recruit suitable participants to form a convenient sample. The inclusion criteria for treatment group participants included: (1) Young people between the ages of 16 and 30, (2) A history of ketamine abuse, (3) Volunteered to attend the 5-day hospitalization program, (4) Uropathy was present and was qualified for short-term treatment by a medical specialist. The exclusion criteria were dual diagnosis of schizophrenia, intellectual disabilities, or organic brain disorders. Social service and drug rehabilitation agencies helped to recruit participants for the comparison group. All the participants in the comparison group were aged 16 to 45, had a history of ketamine abuse or were currently ketamine users, and were not actively involved in drug abuse treatment during the period of assessment and re-assessment. We did not screen if the participants of comparison group had uropathy, as they only attend regular follow-up at the hospital or maintained contact with the community rehabilitation or social services.

We made several assumptions to estimate the sample size required: (1) Need to reach the power of 0.90, (2) α is.05, (3) Medium effect size (Cohen's d) of 0.4, (4) Unequal sample size allocation of 0.3 (treatment vs. comparison), and (5) The design uses repeated measures analysis with one between-subject variable and one within subject variable (at least 2 repeated measures). Using sample size estimation software PASS12 (18), the total sample size required was 75 subjects per group.

### Intervention program

The short-term (5-day) hospitalization program is designed to addresses the key medical and psychosocial issues of ketamine abusers through functional, psychosocial, and lifestyle interventions. During the orientation and the first day of hospitalization, the patient participates in the cognitive (e.g., memory, attention) and motor function (e.g., dexterity, eye-hand-foot coordinator) assessments (19), as well as psychoeducation sessions on how Ketamine abuse may be linked to functional abilities. The assessment reports provided a basis for discussion during motivational interviewing with the patients.

During the next 4 days, patients join the psychosocial group and lifestyle interventions. The psychosocial group focus on exploring how their stress and emotions in daily life are linked to substance use. The group would share on ways to cope with the urge for substance use by using different methods of coping and interpersonal strategies. The therapist will teach common coping strategies (like mindfulness, impulse control, and assertiveness) and practice with participants. The lifestyle re-design group focus on exploring the balance of work, play, self-care, and sleep among participants. The group facilitator will coach the participants to identify daily activities that could promote their mastery and meaning, and set short-term goals on engaging in these activities during the day.

### Instruments

#### Demographic data including age, structured interviews were conducted

We obtained demographic data including age, gender, diagnoses, and educational level when the participants joined the program. We used several standardized instruments to evaluate progress and outcomes from the program. The primary outcome measures included drug use, treatment motivation, and stage of change. The secondary measures included self-efficacy in avoiding drugs, emotions (stress, anxiety, and depression), and lifestyle changes. Data on the measures were collected at different points on the timeline, which included pre-, post-5 days of hospitalization, post-2 weeks, and post-13 weeks. We obtained a minimum of two repeated measures for each outcome variable for the outcome evaluation.

#### Drug use

This was a self-administered questionnaire on the frequency and duration of drug use during the past 3 months. It was a questionnaire developed for evaluation of a drug prevention project called Project Astro Mind (20, 21) and was adopted by the Beat Drugs Fund as one of the standardized questionnaires for reporting program evaluation results. The questionnaire asks participants to report on their frequency of substance use in the past 3 months.

#### Treatment motivation

We used the Texas Christian University (TCU) Scales developed by the Institute of Behavioral Research, for assessing the treatment motivation of participants (22, 23). Participants responded to the questionnaire using a five-point Likert scale, ranging from “strongly disagree” to “strongly agree.” The scale consisted of five subscales reflecting aspects of motivation, namely Problem Recognition (PR), Desire for Help (DH), Treatment Readiness (TR), Pressures for Treatment (PT), and Treatment Needs (TN), representing different dimensions of motivation for treatment. An observational version of the TCU scales demonstrated adequate reliability in a validation study (24), and an interview version of the TCU scales had also been used to predict therapeutic engagement in substance abuse treatment (25).

#### Stage of change

We used the “Contemplation Ladder” to provide a brief self-report measure of motivation to change behavior in substance use (26, 27). Participants were requested to indicate on a 0 (No thought about quitting. I cannot live without drugs) to 10 (I have changed my drug use and will never go back to the way I used drugs before) rating scale, which is anchored to different statements reflecting different stages of readiness for change. Five statements are anchored at items 0, 2, 5, 8, and 10 for reference by respondents. The response option (0) corresponded with the stage of pre-contemplation, (4) to (6) represented the stage of contemplation, (7) and (8) referred to the stage of preparation, and (9) and (10) represented the stages of action and maintenance respectively. Several studies showed that the Contemplation Ladder has good discriminant, convergent, and predictive validity (28) and is closely linked to the stages of change measured by the University of Rhode Island Change Assessment (URICA) (29).

#### Stress, anxiety, and depression

The intervention program aims to reduce stress, anxiety, and depression in the participants. We adopted the stress and anxiety subscales (total of 14 items) of the Chinese version of the Depression Anxiety Stress Scale (DASS21) for monitoring the outcomes. The DASS21 is a 21-item self-report instrument measuring current (over the past week) symptoms of depression, anxiety, and stress (30). The DASS21 had adequate to good internal consistency, and Cronbach's α for the subscale were.84 for the depression subscale, 77 for the anxiety subscale, and.86 for the stress subscale (31). Moussa et al. (32) translated the DASS21 into Chinese and reported that the Chinese version of DASS21 was sensitive to cultural and linguistic issues and could significantly discriminate between the negative emotional syndromes of depression, anxiety, and stress in Chinese populations.

#### The montreal cognitive assessment (MoCA)

The MoCA is designed for screening of mild cognitive impairment linked to early signs of dementia (33), and a Chinese version is used in this study (34). It is also applied in the rapid screening of cognitive ability of people with substance misuse, with acceptable sensitivity (83.3%) and specificity (72.9%) in screening cognitive impairment (35). Using a cutoff of 25 on the MoCA, the overall agreement was 75.0%, and the area under the ROC curve was 0.86.

#### Lifestyle

The FANTASTIC checklist is designed to help health care workers know more about patients' lifestyle when undertaking health care counseling (36, 37). Studies have found that it offers a reliable and quick screening process of lifestyle and is helpful in planning strategies for change (38). In this study, it was used to monitor patients' lifestyle changes that were considered important to health.

#### DASES

The Chinese version of Drug Avoidance Self-Efficacy Scale (DASES) aims to assess self-efficacy in coping with risk situations without using drugs (39). The DASES consists of 16 items specifically concerned with the avoidance of drug use. The questionnaire requests respondents to imagine themselves in situations in which they might be at risk of using drugs. Responses are rated on a 7-point scale ranging from “certainly yes” to “certainly no” which corresponds to a measure of “strength” of self-efficacy. The DASES has been adapted culturally and is a valid and reliable measure of substance users' perceptions of avoidance of self-efficacy (40). It has been adopted as a standardized outcome measure of projects funded by the Beat Drugs Fund of Hong Kong and can be used to monitor treatment progress regarding coping with risk situations in drug use.

### Procedures

Before the start of the study, ethics approval to conduct the study was obtained from the service agencies. Using the Research Information Sheet, research assistants explained to potential subjects the purpose and procedures for the study in a briefing session. Subjects who were willing to participate were requested to sign the consent form for the study. We then conducted preliminary screening of the patients, according to the selection and exclusion criteria, to determine whether the participants should join the treatment or comparison groups.

## Results

There were 84 patients in the treatment group, and 34 participants in the comparison group (Table 1). There was no significant difference in mean age between the treatment (*M* = 27.9, *SD* = 4.81) and comparison groups (*M* = 27.6, *SD* = 5.8) (*t* = 0.37, *p* = 0.73), and the proportion of males and females in both groups was the same (χ^2^ = 2.82, *p* = 0.10). Half (50%) of the participants had completed junior secondary education, and 48.3% had completed senior secondary education. Around half (51.7%) of the participants were employed, and 44.9% were not employed.

**Table 1:** Demographic profile of participants.

**Variable**	**Categories**	**Treatment (*n* = 84)**	**Comparison (*n* = 34)**	**Total (*N* = 118)**
Age	–	M (SD)	M (SD)	M(SD)
		27.9 (4.81)	27.6 (5.78)	27.99 (5.08)
		**n (%)**	**n (%)**	**N (%)**
Sex	Male	36 (42.9%)	18 (52.9%)	54 (45.8%)
	Female	48 (57.1%)	16 (47.1%)	64 (54.2%)
Education	Junior Secondary	47 (56%)	12 (35.3%)	59 (50.0%)
	Senior Secondary	35 (41.6%)	22 (64.7%)	57 (48.3%)
	Post -Secondary	2 (2.4%)	0 (0.0%)	2 (1.7%)
Work status	Not Employed	36 (42.9%)	17 (50.0%)	53 (44.9%)
	Homemaker	2 (2.4%)	1 (3.0%)	3 (2.5%)
	Student	1 (1.1%)	0 (0.0%)	1 (0.8%)
	Employed	45 (53.6%)	16 (47%)	61 (51.7%)

First, we examined the changes in the DASS, TCU Treatment Motivation scales, and the Contemplation Ladder of the treatment group over repeated measures (Table 2). Paired *t*-test results showed that there were significant reductions in drug use between the baseline and the post 13-week measure (*t* = 5.79, *p* < 0.001). We obtained the TCU subscales and Contemplation Ladder measures four times, at pretest, 5 days after hospitalization, and 2 weeks and 13 weeks after hospitalization. Repeated measures ANOVA results showed that there were significant increases in motivation for change (Contemplation Ladder scores) over the four measures (*F* = 56.53, *p* > 0.001), and there was a significant contrast in motivation between pre-test and post 5-day measures. There were significant decreases in three out of the five TCU subscales including problem recognition (*p* < 0.001), desire for help (*p* < 0.001), and pressure for treatment (*p* = 0.05) subscales.

**Table 2:** Change of key outcomes for participants in the treatment group (*n* = 84).

**Outcome measures**	**Time of assessment**				**F or t**	***p***	**$\eta_{\text{p}}^{2}$**
	**Pre-test**	**Post 5 days**	**Post 2 weeks**	**Post 13 weeks**			
	**M (SD)**	**M (SD)**	**M (SD)**	**M (SD)**			
Drug Use (n = 84)	874.25 (1035.53)	–	–	192.27 (309.42)	5.79	0.001>	–
Contemplation Ladder (*n* = 62)	6.63 (0.17)	8.36 (1.08)	8.59 (0.14)	8.58 (0.15)	56.53	0.001>	0.48
**TREATMENT MOTIVATION (*N* = 59)**							
Problem Recognition	4.13 (0.07)	3.99 (0.07)	3.86 (0.07)	3.86 (0.06)	8.95	0.001>	0.13
Desire for Help	4.27 (0.07)	4.22 (0.08)	4.02 (0.08)	3.98 (0.07)	7.37	0.001>	0.11
Treatment Readiness	4.13 (0.07)	4.02 (0.07)	3.88 (0.07)	4.00 (0.08)	3.47	0.02	0.06
Pressure for Treatment	3.57 (0.06)	3.70 (0.11)	3.48 (0.07)	3.43 (0.07)	3.18	0.05	0.05
Treatment Needs	3.67 (0.10)	3.62 (0.09)	3.57 (0.10)	3.53 (0.10)	1.14	0.33	0.02
Cognitive Screening test (MoCA) (n = 47)	25.02 (2.64)	–	–	26.60 (2.75)	4.34	0.001>	–
Lifestyle (FANTASTIC checklist) (n = 83)	1.09 (0.25)	–	–	1.36 (0.61)	3.77	0.001>	–
Drug Avoidance (DASES) (n = 81)	3.82 (1.37)	–	–	3.04 (1.43)	4.67	0.001>	–
**DASS (*N* = 44)**							
Depression	1.22 (0.13)	–	0.86 (0.14)	0.96 (0.15)	3.67	0.04	0.08
Anxiety	1.25 (0.12)	–	0.93 (0.13)	0.91 (0.12)	5.82	0.001	0.12
Stress	1.41 (0.12)	–	1.06 (0.13)	1.14 (0.12)	4.43	0.02	0.09

*$\eta_{p}^{\text{2}}$ is partial Eta-squared*.

For the secondary outcome measures, there were significant increases in the MoCA (*t* = 4.34, *p* < 0.001), lifestyle scores (*t* = 3.77, *p* < 0.001), and self-efficacy in drug avoidance (*t* = 4.67, *p* < 0.001). There were also significant decreases in all subscales of the DASS including the depression (*p* = 0.04), anxiety (*p* = 0.01), and stress (*p* = 0.02) subscales, over the three repeated measures.

Second, we compared the outcomes of the 87 participants in the treatment group with the 34 participants in the control group. Analysis of covariance (ANCOVA) were conducted with pre-test as the covariate and the post-13th-week measure as the outcome (Table 3). This served to equate the large differences in pre-test scores between groups, to enable a more powerful comparison of post-test outcomes. When compared with the comparison group, the treatment group had a decrease in drug use which was close to statistical significance (*p* = 0.07). The treatment group also had a significant decrease in problem recognition (*p* = 0.01), desire for help (*p* < 0.001), and treatment readiness subscales of the TCU scale, and a significant drop in the anxiety subscale of the DASS (*p* = 0.04).

**Table 3:** Comparison of Outcome between Experimental (*n* = 84) and Comparison (*n* = 34) groups using Analysis of Covariance.

**Outcome measure**	***n***		**Post-13 weeks estimated marginal means**		**F**	***p***	**$\eta_{\text{p}}^{2}$**	**Power**
	**Experimental**	**Comparison**	**Experimental**	**Control**				
			**M (SD)**	**M (SD)**				
Drug Use	84	34	192.47 (34.47)	70.96 (55.03)	3.40	0.07	0.03	0.49
Contemplation Ladder	80	32	8.54 (0.16)	8.37 (0.26)	0.29	0.59	0.00	0.08
**TREATMENT MOTIVATION**								
PR	84	34	3.79 (0.06)	3.48 (0.10)	6.30	0.01	0.05	0.70
DH	84	34	3.88 (0.06)	3.51 (0.10)	9.52	0.00	0.08	0.86
TR	84	34	3.97 (0.13)	3.47 (0.21)	4.00	0.05	0.03	0.51
PT	84	34	3.43 (0.06)	3.33 (0.10)	0.82	0.37	0.01	0.15
TN	84	34	3.38 (0.07)	3.45 (0.11)	0.25	0.62	0.00	0.08
**DASS**								
Depression	82	30	0.91 (0.09)	1.05 (0.15)	0.65	0.42	0.01	0.13
Anxiety	82	30	0.94 (0.08)	1.27 (0.13)	4.91	0.03	0.04	0.59
Stress	82	30	1.15 (0.08)	1.25 (0.13)	0.43	0.52	0.00	0.1
FANTASTIC	84	30	1.36 (0.06)	1.23 (0.10)	1.21	0.27	0.01	0.19
DASES	82	27	2.95 (0.14)	2.66 (0.25)	0.93	0.34	0.01	0.16

*PR, Problem Recognition subscale; DH, Desire for Help; TR, Treatment Readiness; PT, Pressure for Treatment; TN, Treatment Needs; DASS, Depression Anxiety Stress Scales; FANTASTIC is a lifestyle scale, DASES, Drug Avoidance Self-Efficacy Scale subscales. $\eta_{p}^{\text{2}}$ is partial Eta-squared*.

We collected feedback from both the patients and the NGO case social workers. Both the patients and social workers reported very positive experiences about the program. Clinically, they regarded the presentation of clinical assessment results to the patients, followed by brief motivational interviewing as key factors leading to the success of the program.

## Discussion

The results from the outcome evaluation suggest that participants in the treatment group made progress in their stage of change. On average, participants moved from pre-contemplation to the stage of contemplation or preparation. This point is evident from the positive changes in the Contemplation Ladder. Most patients reached the stage of contemplation or preparation after the 5-day hospitalization program and then continued to stay in these stages of change after discharge. They also showed significant decreases in their drug use, higher self-efficacy to avoid drug use, and a perceived lower need for more treatment. The positive changes were also reflected in the changes in the secondary outcome measures including improvement of cognitive ability and reduction of negative emotions. These illustrate the positive achievements of the program over the 5-day hospitalization program, as well as during the follow-up period of 13 weeks.

We also conducted repeated measures ANOVA to compare the outcome profile of the treatment group with the comparison group. When set alongside the comparison group, the treatment group had substantial decreases in negative emotions (especially anxiety) and decreases in treatment needs (on three out of five subscales), and they made progress in their stage of change from pre-contemplation toward the stage of preparation. However, there were no significant changes in the lifestyle scale (*p* = 0.27) or in self-efficacy in avoidance of drug use (*p* = 0.34). Part of the reason for the less positive comparison result could be attributed to the rehabilitation status of the participants in the comparison group. Many comparison group participants had very low drug use frequencies, and very high scores in the Contemplation Ladder at the baseline assessment. This indicates that many control group participants had stopped or were close to stop taking drugs, and some were engaged in part-time jobs, job retraining, or were volunteers in drug rehabilitation programs. The outcome measures for many of the comparison group participants were close to the ceiling and remained unchanged over the 13 weeks. When these participants were compared with the treatment group who were active drug abusers, it may not reflect any significant progress for the treatment group.

Considering both the outcome changes of the experimental group over three to four repeated measures, and a comparison with the control group, we found partial support for the program. While the treatment group participants had significant positive changes, the results looked less strong when they were compared with the comparison group.

There are several limitations to this study. First, it was a great challenge to collect a range of drug rehabilitation outcomes repeatedly from treatment group participants, especially after they were discharged from hospital. Although we managed to obtain a sample size that ensured a powerful analysis, the amount of missing data was large and could have led to some bias in the interpretation of results. We met the sample size requirement for the treatment group, but not for the control group. It was harder than expected to recruit control group participants. Many people who recovered from drug abuse declined to participate as they are engaged in study or work during the day. Others tend to avoid revealing their status of drug abuse after they were discharged from services. Around 25% of participants in control only completed the first assessment and missed the re-assessment, resulting in data loss for control group.

Second, both the treatment group participants and the social workers found the cognitive and functional assessments, together with motivational interviewing, were most beneficial. The 5-day in-patient program also contained other elements (like an emotions management program and relapse prevention strategies), which we could not evaluate separately. We obtained reports from social service agencies about the follow-up outcomes of the participants, but we understand that there could have been some variation in the type and form of action and social support in the community. We do not have full information to analyze how the community-based interventions and support may help to maintain the positive outcomes in the treatment group. Third, we would need to consider adding some specific criteria in the future recruitment of a comparison group. We could exclude those who have stopped taking drugs completely or have quite fully engaged in work or education. These participants would likely report an outcome profile (such as drug use) that does not change over time.

Overall, the study is an important addition to the current literature. First, the CAP program is one of the few treatment programs addressing the needs of ketamine abusers who seek help for urological and medical problems in emergency rooms (6, 41). The program successfully engaged many patients for short-term hospitalization, during which time psychosocial interventions could address their ketamine addiction. The results implied that clinician should regularly screen for urological problems in Ketamine users during medical consultations, as it could provide an opportunity for engaging the patient in managing their substance misuse. Second, the short-term hospitalization program, in collaboration with social service agencies, provides a partnership model that is essential for success in drug rehabilitation programs. Third, the CAP uses a community and internet out-reach and short-term treatment approach that addresses successfully the engagement of many “hidden youths” who are disengaged from social institutions. Fourth, the study results provide valuable research evidence in the treatment of ketamine abusers that is largely lacking in the current literature (42). Few previous studies have been able to recruit a comparison or control group, to conduct partnership programs between hospital and the community, or to continue to monitor the change of drug-related outcomes over time. Despite the challenges in recruitment of participants and the implementation of repeated measures, the study could recruit a sizeable group of participants that provided adequate power for the evaluation of a non-equivalent group design.

Overall, the CAP is effective in reducing drug use and anxiety and helps patients to move from pre-contemplation to the contemplation or preparation stages of change. The study results underscore a new approach in mental health services to engaging young people who abuse ketamine. Young people who seek urgent medical attention for urological and medical problems are receptive to psychosocial interventions that help them manage their addiction. A package of interventions covering motivational interviewing, emotions management, and lifestyle re-design could make an impact on motivation for change and reducing drug use. The combination of short-term hospitalization and community support and social work services, is essential to the success in the management of young people with ketamine abuse problems.

## Ethics statement

This study was carried out in accordance with the recommendations of ethical guidelines, Joint Chinese University of Hong Kong - New Territories East Cluster (CUHK-NTEC) Clinical Research Ethics Committee. The research protocol was approved by the Joint CUHK-NTEC Clinical Research Ethics Committee. All subjects gave written informed consent in accordance with the Declaration of Helsinki.

## Author contributions

AS designed the research study and conducted the research data collection and data analysis. He was also the key person in preparing and writing the research report and journal manuscript. FK designed the psychosocial and occupational therapy interventions of the CAP, and led a team of health professionals in implementing the Program. SM is a urological specialist who provides medical and specialist care to young people who abuse ketamine. He also screened, assessed, and provided treatment to patients and made referrals of these patients to the CAP.

### Conflict of interest statement

The authors declare that the research was conducted in the absence of any commercial or financial relationships that could be construed as a potential conflict of interest.

## References

[1] Narcotics Division, Security, Bureau Central Registry of Drug Abuse Drug Statistics 2015. Available online at: http://www.nd.gov.hk/statistics_list/doc/tc/t3.pdf (2015) (Accessed January, 2018).

[2] Narcotics Division Security Bureau CRDA Drug Abuse Statistics. Available online at: https://www.nd.gov.hk/en/statistics_list.htm (2016). (Accessed July 5, 2018).

[3] Chan KW Lee TM Siu AM Wong DP Kam CM Tsang SK. Effects of chronic ketamine use on frontal and medial temporal cognition. Addict. Behav. (2013) 38:2128–32. 10.1016/j.addbeh.2013.01.014 23435274

[4] Copeland J Dillon P The health and psycho-social consequences of ketamine use. Int J Drug Policy (2005) 16:122–31. 10.1016/j.drugpo.2004.12.003

[5] Liang HJ Tang KL Chan F Ungvari GS Tang WK Ketamine users have high rates of psychosis and/or depression. J Addict Nurs. (2015) 26:8–13. 10.1097/JAN.0000000000000060 25761158

[6] Morgan CJ Curran HV. Ketamine use: a review. Addiction (2012) 107:27–38. 10.1111/j.1360-0443.2011.03576.x 21777321

[7] Mak SK Chan MTY Bower WF Yip SKH Hou SSM Wu BBB. Lower urinary tract changes in young adults using ketamine. J Urol. (2011) 186:610–4. 10.1016/j.juro.2011.03.108 21684556

[8] Middela S Pearce I. Ketamine-induced vesicopathy: a literature review. Int J Clin Pract. (2011) 65:27–30. 10.1111/j.1742-1241.2010.02502.x 21155941

[9] Tam YH Ng CF Pang KKY Yee CH Chu WCW Leung VYF. One-stop clinic for ketamine-associated uropathy: report on service delivery model, patients' characteristics and non-invasive investigations at baseline by a cross-sectional study in a prospective cohort of 318 teenagers and young adults. BJU Int. (2014) 114:754–60. 10.1111/bju.12675 24552244

[10] Wood D Cottrell A Baker SC Southgate J Harris M Fulford S. Recreational ketamine: from pleasure to pain. BJU Int. (2011) 107:1881–4. 10.1111/j.1464-410X.2010.10031.x 21314885

[11] Cheung C Acute and chronic toxicity pattern in ketamine abusers in Hong Kong. J Med Toxicol. (2012) 8:267–270. 10.1007/s13181-012-0229-z 22552737 PMC3550162

[12] Robles-Martínez M Abad AC Pérez-Rodríguez V Ros-Cucurull E Esojo A Roncero C. Delayed Urinary Symptoms Induced by Ketamine. J. Psychoactive Drugs. (2017) 50:129–32. 10.1080/02791072.2017.1371364 28934076

[13] Chu PSK Ma WK Wong SCW Chu RWH Cheng CH Wong S. The destruction of the lower urinary tract by ketamine abuse: a new syndrome? BJU Int. (2008) 102:1616–22. 10.1111/j.1464-410X.2008.07920.x 18680495

[14] D'Amico EJ Miles JN Stern SA Meredith LS. Brief motivational interviewing for teens at risk of substance use consequences: a randomized pilot study in a primary care clinic. J Subst Abuse Treat. (2008) 35:53–61. 10.1016/j.jsat.2007.08.008 18037603

[15] Rollnick S Miller WR Butler CC Aloia MS Motivational interviewing in health care: helping patients change behavior. New York, NY: Guildford Press (2008).

[16] Eklund M Leufstadius C Bejerholm U. Time use among people with psychiatric disabilities: Implications for practice. Psychiatr Rehabil J. (2009) 32:177–91. 10.2975/32.3.2009.177.191 19136350

[17] DiClemente CC Schlundt D Gemmell L. Readiness and stages of change in addiction treatment. Am J Addict (2004) 13:103–19. 10.1080/10550490490435777 15204662

[18] Hintze J PASS 12. Kaysville: NCSS, LLC Available online at: www.ncss.com (2013).

[19] Ruff RM Parker SB. Gender and age-specific changes in motor speed and eye-hand coordination in adults: normative values for the finger tapping and Grooved Pegboard Tests. Percept Motor Skills (1993) 76:1219–30 10.2466/pms.1993.76.3c.1219 8337069

[20] Lam CW Shek DT Ng HY Yeung KC Lam DO. An innovation in drug prevention programs for adolescents: the Hong Kong Astro Project. Int J Adolesc Med Health (2011) 17:343–54. 10.1515/IJAMH.2005.17.4.343 16445073

[21] Shek DTL A Longitudinal Evaluation Study of a Pioneering Drug Prevention Program (Project Astro MIND) in Hong Kong. Hong Kong: Beat Drugs Fund Association (2003).

[22] Knight K Holcom M Simpson DD TCU Psychosocial Functional and Motivation Scales: Manual on Psychometric Properties. Fort Worth: Texas Christian University, Institute of Behavioral Research (1994).

[23] Simpson DD Joe GW Knight K Rowan-Szal G.A Gray JS. Texas Christian University (TCU) short forms for assessing client needs and functioning in addiction treatment. J. Offender Rehabil. (2012) 51:34–56. 10.1080/10509674.2012.633024 22505795 PMC3325103

[24] Gongora VC DeWeert-van Oene GH von Sternberg K de Jong CA Validation of the observational version of the motivation for treatment scale. Addict Res Theory. (2012) 20:414–22. 10.3109/16066359.2012.656757

[25] Hiller ML Knight K Leukefeld C Simpson DD Motivation as a predictor of therapeutic engagement in mandated residential substance abuse treatment. Crim Justice Behav. (2002) 29:56–75. 10.1177/0093854802029001004

[26] Biener L Abrams DB. The contemplation ladder: validation of a measure of to consider smoking cessation. Health Psychol. (1991) 10:360–5. 10.1037/0278-6133.10.5.360 1935872

[27] Slavet JD Stein LAR Colby SM Barnett NP Monti PM Golembeske C. The marijuana ladder: Measuring motivation to change marijuana use in incarcerated adolescents. Drug Alcohol Depend. (2006) 83:42–48. 10.1016/j.drugalcdep.2005.10.007 16289930 PMC2754131

[28] Hogue A Dauber S Morgenstern J. Validation of a contemplation ladder in an adult substance use disorder sample. Psychol Addict Behav. (2010) 24:137–44. 10.1037/a0017895 20307121 PMC2845310

[29] Amodei N Lamb RJ. Convergent and concurrent validity of the Contemplation Ladder and URICA scales. Drug Alcohol Depend. (2004) 73:301–6. 10.1016/j.drugalcdep.2003.11.005 15036552

[30] Lovibond SH Lovibond PF Manual for the Depression Anxiety Stress Scales. 2nd edn. Sydney: Psychology Foundation (1995).

[31] Henry JD Crawford JR. The short-form version of the Depression Anxiety Stress Scales (DASS-21): Construct validity and normative data in a large non-clinical sample. Br J Clin Psychol. (2005) 44:227–39. 10.1348/014466505X29657 16004657

[32] Moussa MT Lovibond PF Laube R Psychometric Properties of a Chinese Version of the Short Depression Anxiety Stress Scales (DASS21). Report for New South Wales Transcultural Mental Health Centre. Sydney, NSW: Cumberland Hospita (2001).

[33] Nasreddine ZS Phillips NA Bédirian V Charbonneau S Whitehead V Collin I. The Montreal Cognitive Assessment, MoCA: a brief screening tool for mild cognitive impairment. J Am Geriatr Soc. (2005) 53:695–9. 10.1111/j.1532-5415.2005.53221.x 15817019

[34] Wong A Xiong YY Kwan PW Chan AY Lam WW Wang K. The validity, reliability and clinical utility of the Hong Kong Montreal Cognitive Assessment (HK-MoCA) in patients with cerebral small vessel disease. Dement Geriatr Cogn Disord. (2009) 28:81–7. 10.1159/000232589 19672065

[35] Copersino ML Fals-Stewart W Fitzmaurice G Schretlen DJ Sokoloff J Weiss RD. Rapid cognitive screening of patients with substance use disorders. Exp Clin Psychopharmacol. (2009) 17:337–44. 10.1037/a0017260 19803633 PMC3144764

[36] Douglas W Ciliska D Lifestyle assessment: development and use of the fantastic checklist. Can Family Physician (1984) 30:1527–32.

[37] Douglas MCW The fantastic lifestyle checklist: design and application. Recreat Res Rev. (1984) 11:17–21.

[38] Wilhelm K Handley T Reddy P. Exploring the validity of the Fantastic Lifestyle Checklist in an inner city population of people presenting with suicidal behaviours. Austr N Z J Psychiatry (2016) 50:128–34. 10.1177/0004867415621393 26681263

[39] Martin GQ Wilkinson D.A Poulos CX. The drug avoidance self-efficacy scale. J Subst Abuse. (1995) 7:151–63. 7580226 10.1016/0899-3289(95)90001-2

[40] Norozi E Miri MR Soltani R Eslami AA Harivandi AR Dastjerdi R Drug Avoidance Self Efficacy Scale (DASES): a cultural adaptation and validation study. J Subst Use. (2016) 21:449–54. 10.3109/14659891.2015.1056851

[41] Wang YC Chen SK Lin CM Breaking the drug addiction cycle is not easy in ketamine abusers. Int J Urol. (2010) 17:496 10.1111/j.1442-2042.2010.02491.x 20415711

[42] Shek DT Tackling adolescent substance abuse in Hong Kong: where we should and should not go. Sci World J. (2007) 7:2021–30. 10.1100/tsw.2007.315 PMC5901312 18167617

