# Corrigendum: Outcome Evaluation of a Short-Term Hospitalization and Community Support Program for People Who Abuse Ketamine

**DOI:** 10.3389/fpsyt.2018.00746

**Published:** 2019-01-22

**Authors:** Frontiers Editorial Office

**Affiliations:** Frontiers Media SA, Lausanne, Switzerland

**Keywords:** ketamine, drug misuse, outcome, evaluation, intervention, hospitalization, Chinese

In the original article, there was an error as one of the reviewers did not endorse the final version of the article. This correction is made to remove this individual as an endorsing reviewer on the article. The final, published article has been endorsed by the Specialty Chief Editor, the Associate Editor and the remaining reviewer. The original article has been updated.

